# Effect of Popping and Steam Cooking on Total Ferulic Acid, Phenolic and Flavonoid Contents, and Antioxidant Properties of Sukhothai Fragrant Black Rice

**DOI:** 10.3390/foods15020320

**Published:** 2026-01-15

**Authors:** Thayada Phimphilai, Onsaya Kerdto, Kajorndaj Phimphilai, Phronpawee Srichomphoo, Wachiraporn Tipsuwan, Pornpailin Suwanpitak, Yanping Zhong, Somdet Srichairatanakool

**Affiliations:** 1 Chiang Mai University Demonstration School, Faculty of Education, Chiang Mai University, Chiang Mai 50200, Thailand; thayada.nene@gmail.com (T.P.); pornpailin.s@cmu.ac.th (P.S.); 2 Department of Biochemistry, Faculty of Medicine, Chiang Mai University, Chiang Mai 50200, Thailand; onsaya35@gmail.com (O.K.); yanping_z@cmu.ac.th (Y.Z.); 3 Department of Mechanical Engineering, Faculty of Engineering, Chiang Mai University, Chiang Mai 50200, Thailand; kajorndej.p@cmu.ac.th; 4 Research Excellence, School of Medicine, University of Phayao, Phayao 56000, Thailand; phronpawee0402@gmail.com; 5 Division of Biochemistry, School of Medical Sciences, University of Phayao, Phayao 56000, Thailand; wachiraporn.ti@up.ac.th; 6 School of Medical Technology and Artificial Intelligence, Youjiang Medical University for Nationalities, Baise 533000, China

**Keywords:** rice, *Oryza sativa*, antioxidant, ferulic acid, phenolic, popping, cytotoxicity

## Abstract

This study investigated the effects of thermal processing and extraction solvents on the phytochemical composition, antioxidant potential, and cytotoxic activity of Sukhothai fragrant rice (*Oryza sativa* L.). Rice subjected to three processing methods, unprocessed (raw), popped/puffed and steam-cooked, was extracted using hot water or 70% (*v*/*v*) ethanol, yielding six extracts. Trans-ferulic acid, γ-oryzanol and anthocyanins were quantified using HPLC-DAD and HPLC-ESI-MS, while total phenolic and flavonoid contents, and antioxidant activities were evaluated using Folin–Ciocalteu, aluminium chloride, DPPH and ABTS assays. Cytotoxicity was assessed in Huh7 hepatocellular carcinoma cells. Water extracts consistently produced higher yields and contained greater total phenolic, flavonoid and anthocyanin contents, resulting in stronger antioxidant activity. Unprocessed rice water extract exhibited the highest trans-ferulic acid recovery and antioxidant capacity. Thermal processing, particularly steamed cooking, markedly reduced phytochemical contents, likely due to heat-induced degradation. In contrast, ethanolic extracts yielded lower quantities but higher concentrations of less polar bioactive compounds and exhibited greater cytotoxic effects. Overall, minimal thermal processing combined with aqueous extraction best preserved antioxidant compounds, while ethanolic extraction enhanced biological potency. These findings highlight the importance of processing intensity and solvent polarity in optimizing the nutraceutical and functional potential of black rice.

## 1. Introduction

Black rice (*Oryza sativa* L.), also known as forbidden rice, is a pigmented cereal grain recognized for its high nutritional value and abundance of bioactive phytochemicals such as anthocyanins, phenolic acids, flavonoids, and γ-oryzanol [1,2,3]. These compounds contribute significantly to its antioxidant, anti-inflammatory, and chemoprotective properties, which make black rice a promising functional food and nutraceutical ingredient [1,4,5,6]. Among these phytochemicals, ferulic acid is a major hydroxycinnamic acid esterified to the rice bran matrix, playing an important role in scavenging free radicals and protecting against oxidative damage [7]. Notably, the n-butanol extract of black rice leaves contained the highest levels of phenolics, flavonoids, and anthocyanins, and exhibited strong antioxidant activity; in contrast, the ethanolic and ethyl acetate extracts considerably decreased nitric oxide production and suppressed the secretion of inflammatory cytokines including prostaglandin E2, interleukins 6 and interleukin 1β, and tumor necrotic factor in lipopolysaccharide-stimulated RAW264.7 cells [8].

Hom-nin black rice contained significantly higher concentration of γ-oryzanols, phytosterols, tocopherols and tocotrienols than Jasmine red rice and the nonpigmented Thai fragrant Khao Dawk Mali 105 white rice, while rice bran oil obtained by cold-press extraction exhibited higher concentrations of these phytochemicals than oils extracted using supercritical CO_2_ or conventional solvent extraction [9]. However, thermal processing, such as puffing, steaming, and cooking, can substantially alter the chemical stability and extractability of these bioactive phytochemicals. Heat treatment may degrade anthocyanins and phenolic acids through oxidation, polymerization, and complex formations with starch and proteins, ultimately reducing antioxidant potential [6,10]. Conversely, moderate processing can sometimes enhance the release of bound phenolics by disrupting cell walls and promoting solubilization [1,11,12]. Therefore, understanding the effects of specific processing techniques and extraction solvents is essential for optimizing the recovery of functional compounds from black rice. Popping/puffing methods are traditionally practiced to improve the storage and shelf life, increase organoleptic properties, and to more easily incorporate certain grains into ready-to-eat-foods. Among them, electric popping/puffing machines involve conduction of heat and mass transfer for continuous fluidization [13].

Sukhothai fragrant black rice is a regional-specific Thai rice variety that is primarily cultivated in the Sukhothai Province of Thailand. It is a Thai crossbreed of black jasmine rice (Hom Nin strain) and white jasmine rice (Khao Dawk Mali 105 strain) and has been known for its deep purple color, soft and sticky texture, aromatic quality and soft, slightly sticky texture after cooking. Interestingly, it contains elevated levels of phenolic acids, particularly trans-ferulic acid, as well as flavonoids, anthocyanins, γ-oryzanol, and vitamin E homologues, compounds that have been associated with antioxidant, anti-inflammatory, and chemoprotective activities. The present study aimed to comparatively investigate the impact of popping and steam cooking, in combination of hot-water and 70% ethanol extraction, on the phytochemical composition, antioxidant activities, and cytotoxic potential of *Sukhothai fragrant black rice*.

## 2. Materials and Methods

### 2.1. Chemicals and Reagents

2,2-Azino-*bis*-(3-methylbenzothiazoline-6-sulfonic acid (ABTS) (Product number A1888, >98% pure), aluminum chloride anhydrous powder (Product number 563919, 99.99% pure), 2,2-diphenyl-1-picrylhydrazyl (DPPH) (Product number D9132, >95% pure), 3-(4,5-dimethylthiazol-2-yl)-2,5-diphenyltetrazolium bromide (MTT) (Product number 475989), 6-hydroxy-2,5,7,8-tetramethylchroman-2-carboxylic acid (Trolox) (Product number 238813, 97% pure), potassium acetate (Product number 236497, >99% pure), sodium carbonate (Product number S2127, >99.5% pure), quercetin (Q) (Product number Q4951, 95% pure), 3,4,5-trihydroxybenzoic acid or gallic acid (GA) (Product number G7384, 97.5–102.5% pure), and trans-ferulic acid (Product number W518301, >99% pure) were purchased from Sigma-Aldrich Chemicals Company Limited (Saint Louis, MO, USA). Dimethyl sulfoxide (DMSO) and potassium persulfate (Product number 216224) were purchased from Merck KGaA, Darmstadt, Germany. Folin–Ciocalteu’s phenol reagent (Catalog number J-4100-08) and Dulbecco’s modified Eagle medium (DMEM) (Catalog number 11965092) were purchased from Thermo Fisher Scientific, Middlesex Counter, MA, USA. Glacial acetic acid (Catalogue number CABDH30983, 99.7–100.5% pure), acetonitrile (Catalogue number BDH83639, >99.9% pure), ethyl acetate (Catalogue number BDH1123, >99.5% pure), and 70% (*v*/*v*) ethanol (>96% pure) were purchased from BDH Company, West Yorkshire, England. All organic solvents were of the highest pure Analytical or HPLC grade.

### 2.2. Black Rice Samples

#### 2.2.1. Raw Rice

De-husked unpolished black fragrant rice (brand name: Khao Hom Sukho, Premium grade) was obtained from Organic Agriculture Project, Sukhothai Airport, Dhammachart Na Thai Company Limited, Sawankhalok, Sukhothal, Thailand). Rice (300 g) was rinsed thoroughly twice with deionized water (DI) (500 mL each) and dried in an air-flow oven at 50 °C to remove the excess starch that typically tends to make rice clumpy.

#### 2.2.2. Popping/Puffing Rice

Rice was popped and puffed using the method established by Tavanandi et al. [13] and operated by Phimpilai K. [14]. Firstly, the moisture contents of the grains were adjusted to 13–14% and heated in an expansion chamber (diameter 0.15 m) of a popping/puffing machine (Model: Goldenpack, capacity of 10–20 kg/hour, Siam Golden Group, Bangkok, Thailand) preset at 250 °C. Process parameters, such as fluidization velocity (4.18–5.78 m/s), terminal velocity (6.89 m/s), carry over velocity (2.15–6.18 m/s), convective heat and mass transfer coefficients (103–187 W/m^2^ and 0.124–0.162 m/s, respectively), were estimated for popped/puffed rice. In the procedure, paddy (100 g) was added to the preheated iron roasting pan and popped by continuous stirring under a pressure setting of 0.8–1.5 MPa for 70–80 s. At the end of the puffing process, the chamber was opened, resulting in a sudden drop in pressure, which resulted in the instantaneous evaporation of the water in the grains. The puffed grains were cooled to room temperature, ground using a hand food grinder, and passed through a 250 μm mesh sieve. The flour from the grains was sealed in polyethylene bags and stored in a desiccator until further analysis.

#### 2.2.3. Cooked Rice

The washed rice was weighed to 300 g, put into to a cooking pot, and cooked with 300 mL of DI in an electric rice cooker (Sharp brand, Sharp Corporation Company Limited, Nonthaburi, Thailand) according to the manufacturer’s manual. Once the rice cooking process was finished, the rice was allowed to rest for 5 to 10 min.

### 2.3. Preparation of Rice Extracts

#### 2.3.1. Water Extraction

Uncooked, popped/puffed, and cooked black rice (100 g each) were extracted with 1 L of DI at 80 °C for 10 min. The extracts were then filtered through a double layer of cheesecloth and subsequently centrifuged at 6000 rpm for 20 min at 25 °C. The resulting supernatants were lyophilized overnight to obtain dried extracts. The dried extract powders were then stored in tightly sealed plastic bottles, wrapped in aluminum foil, and kept at −20 °C until further analysis [15].

#### 2.3.2. Ethanolic Extraction

Similarly, the black rice samples (100 g each) were extracted with 1 L of 70% ethanol. The mixtures were filtered through a double layer of cheesecloth and then centrifuged at 6000 rpm for 20 min at 25 °C. The supernatants were concentrated using a rotary evaporator under reduced pressure and subsequently lyophilized overnight to obtain the dried extracts. The resulting extract powders were stored in tightly sealed plastic containers, wrapped in aluminum foil, and kept at −20 °C until further analysis [15].

### 2.4. Chemical Composition Analysis

#### 2.4.1. Total Phenolic Content (TPC)

TPC was determined using the Folin–Ciocalteu colorimetric method. Briefly, 100 μL of GA standard solution or sample extract was mixed with 200 μL of 10% (*v*/*v*) Folin–Ciocalteu reagent in a test tube. Subsequently, 800 μL of 700 mM sodium carbonate solution was added, and the mixture was thoroughly mixed and allowed to stand in the dark at room temperature for 30 min. The optical density (OD) was measured at 765 nm using a double-beam UV–Vis spectrophotometer (Shimadzu Corporation, Nakagyo-ku, Kyoto, Japan). The TPC was quantified from a calibration curve of GA and expressed as mg of gallic acid equivalent/g of extract (mg GAE/g) [16].

#### 2.4.2. Total Flavonoid Content (TFC)

TFC was determined using the aluminum chloride colorimetric method. Briefly, 250 μL of Q standard solution or sample extract was mixed with 50 μL of 10% (*v*/*v*) aluminum chloride solution and 50 μL of 1 M potassium acetate solution in a test tube. Subsequently, 2.15 mL of DI was added, and the mixture was thoroughly mixed and incubated in the dark at room temperature for 30 min. The OD was measured at 415 nm using a UV–Vis spectrophotometer. The TFC was calculated from the Q calibration curve and expressed as mg of quercetin equivalents/g of extract (mg QE/g) [17].

### 2.5. HPLC-DAD Analysis of Total Trans-Ferulic Acid

Total ferulic acid was quantified using the high-performance liquid chromatography-diode array detector (HPLC-DAD) method established by Watanabe et al. and slightly modified by Banchuen et al. [18,19]. In the sample preparation, 0.5 g of rice extract was hydrolyzed with 50 mL of 1 M NaOH for 3 h at 40 °C and neutralized by the addition of 26 mL of 2 M HCl. The sample was partitioned three times with 50 mL of ethyl acetate (5 min for each), and all the ethyl acetate layer was pooled and evaporated. Afterward, the residual was reconstituted with a solvent mixture of methanol and H_2_O (1:1 by volume) and filtered through a syringe-driven polytetrafluoroethylene (PTFE) filter membrane (0.45-μm pore size, 13 mm diameter, Monotaro Company, Limited, Tokyo, Japan) prior to injection. In our analysis, 5-μL aliquot of sample solution was injected into the Agilent HPLC system (Model 1290 Infinity II, Agilent Technologies, Inc., Santa Clara, CA, USA) equipped with a DAD and fractionated on a column (Agilent Eclipse XDB-C18, dimension of 4.6 mm × 250 mm, 5 μm particle size, Agilent Technologies, Inc., Santa Clara, CA, USA) that had been thermoregulated at 40 °C. The analytes were eluted with isocratic mobile-phase solvent comprising 2.5% (*v*/*v*) acetic acid and acetonitrile (88:12, *v*/*v*) at a flow rate of 0.5 mL/min and online detected at a wavelength of 320 nm. Standard ferulic acid was used for the construction of a calibration curve and the identification of ferulic eluent in the sample. In the analysis, the lowest limit of detection (LOD) of ferulic acid was found to be 0.07 μg/L.

### 2.6. HPLC-DAD Analysis of Total γ-Oryzanol

Total γ-oryzanol was quantified using a HPLC-DAD method with slight modification [20,21]. Briefly, 0.05 mg of γ-oryzanol standard and 5.0 mg of rice extract were dissolved separately in 1 mL of a solvent mixture consisting of ethanol and acetonitrile (2:3, *v*/*v*). The solutions were mixed thoroughly, sonicated for 5 min and filtered through a syringe-driven PTFE membrane filter, 0.45-μm pore size, 13 mm diameter) prior to injection. For analysis, a 5-μL aliquot of each sample solution was injected into an Agilent HPLC system (Model 1290 Infinity II, Agilent Technologies, Inc., Santa Clara, CA, USA) equipped with a DAD and fractionated on a column (Agilent Zorbax C18, dimension of 4.6 mm × 150 mm, 5 μm particle size, Agilent Technologies, Inc., Santa Clara, CA, USA) maintained at 40 °C. The analytes were eluted using a linear gradient system with mobile-phase solvents A consisting of 0.1% (*v*/*v*) formic acid in DI water and mobile-phase solvent B consisting of acetonitrile, starting at 1:99 (A:B, *v*/*v*) pattern for 35 min at a flow rate of 1.0 mL/min. Detection was performed at a wavelength of 325 nm. Total γ-oryzanol content in the rice extracts was quantified by comparison with a γ-oryzanol standard (50 μg/mL) and expressed as mg/g of extract and μg/g of rice. The LOD of γ-oryzanol was determined to be 0.05 μg/L.

### 2.7. HPLC-ESI-MS Analysis of Anthocyanins

Anthocyanins in rice extracts were identified and quantified using high-performance liquid chromatography–electrospray ionization–mass spectrometry (HPLC-ESI-MS). The method was adapted from previously established protocols with minor modifications to optimize separation and detection [22,23]. Rice extracts were first reconstituted in 1.0 mL of DI water-acetonitrile (1:1, *v*/*v*) solvent, sonicated for 5 min, and filtered through a syringe PTFE filter membrane prior to analysis. An aliquot of 20 μL was injected into the Agilent HPLC system consisted of a quaternary pump (G1311A), an online vacuum degasser (G1322A), an autosampler (G1313A), a thermostated column compartment (G1316A) and a photodiode array (PDA) detector (G1315A). The PDA outlet was directly coupled to the atmospheric pressure ESI interface of a mass spectrometer (Agilent Technologies 1100 LC/MSD SL, Palo Alto, CA, USA) via a 1:1 flow splitter. Chromatographic separation was performed on a column (Zorbax C18, 150 mm × 4.6 mm, 5 µm particle size; Agilent Technologies Inc., Santa Clara, CA, USA) maintained at 30 °C. The mobile-phases solvents A (0.1% formic acid) and B (acetonitrile) were delivered at a flow rate of 1.0 mL/min using the following gradient program: A:B (93:7, *v*/*v*) ⟶ (80:20, *v*/*v*) for 5 min, followed by re-equilibration for 5 min. Anthocyanins were detected at 520 nm, which is characteristic of these pigments. Mass spectrometric analysis was conducted in positive ESI mode, with spectra acquired over a mass-to-charge ratio (*m*/*z*) range of 100–500. For the single-quadrupole MS system, the ESI energy was set at 70 eV, and the ion source and interface temperatures were maintained at 150 °C and 230 °C, respectively. Nitrogen was used as the nebulizing, drying, and collision gas. The capillary temperature was set to 320 °C, nebulizer pressure to 60 pounds·inch^2^, and drying gas flow rate to 13 L/min. Capillary voltages were set at 4000 V (positive) and 3500 V (negative). The oven temperature program was as follows: 80 °C (3 min hold), ramped to 110 °C at 10 °C/min (5 min hold), increased to 190 °C (3 min hold), ramped to 220 °C at 10 °C/min (4 min hold), and finally increased to 320 °C at 15 °C/min (13 min hold). Accurate mass measurements were obtained using an automatic mass calibration method with an external calibration solution (ESI-L Low Concentration Tuning Mix; Agilent calibration solution B). The LOD and recovery were determined to be 0.2 μg/g and 70–110%, respectively. Chromatographic and mass spectrometric data analysis, including chemical formula prediction and exact mass calculations, were performed using MassHunter software version B.04.00 build 4.0.479.0 (Agilent Technologies Inc., Santa Clara, CA, USA).

### 2.8. Analysis of Antioxidant Activity

#### 2.8.1. ABTS Method

Firstly, ABTS^+^ working solution was freshly prepared by mixing 10 mL of 7 mM ABTS solution with 10 mL of 2.45 mM potassium persulfate solution in a test tube. The mixture was then kept in the dark at room temperature for 18 h to allow for the formation of the ABTS^+^ radical cation, which was indicated by the development of a dark blue color. Prior to use, the OD of the ABTS^+^ working solution was adjusted to 0.70 ± 0.02 at 734 nm using DI. For the assay, 20 μL of each sample concentration (0–40 mg/mL) was mixed with 180 μL of the ABTS^+^ working solution in a 96-well microplate. A blank well was prepared by mixing 180 μL of ABTS^+^ solution with 20 μL of DI. The reaction mixtures were then incubated in the dark at room temperature for 10 min, and the OD was measured at 734 nm using a microplate reader (Synergy H4, BioTek Instruments, Winooski, VT, USA) [24], Accordingly, radical scavenging activity was calculated using Equation (1),

(1) ABTS^•+^ scavenging activity (%) = [(OD_control_ − OD_sample or Trolox_)/OD_control_] × 100

#### 2.8.2. DPPH Assay

The radical scavenging activity of the ginger extract was evaluated using the DPPH assay. Various concentrations of the extract (0–80 mg/mL) and Trolox standard (0.002–0.5 mg/mL) were prepared. A 0.2 mL aliquot of each sample or standard solution was mixed with 0.2 mL of 0.4 mM DPPH^•^ solution. The mixtures were incubated in the dark at room temperature for 30 min. After incubation, OD was measured at 517 nm using a UV-Vis spectrophotometer [25]. The antioxidant activity was expressed as the percentage of DPPH^•^ radical inhibition, as calculated using Equation (2).

(2) Radical scavenging activity (%) = [(OD_control_ − OD_sample or Trolox_)/OD_control_] × 100

### 2.9. Cytotoxicity Test

#### 2.9.1. Huh7 Cell Culture and Treatment

The cytotoxicity of the water extracts obtained from unprocessed, popped/puffed, and cooked rice was evaluated in hepatocytes using the MTT method that involved the mitochondrial reductase enzyme system in viable cells. Human hepatocellular carcinoma (Huh7) cells were cultured in DMEM at 37 °C for 12 h and seeded in 96-well plates at a density of 1 × 10^4^ cells per well and incubated in a humidified CO_2_ incubator at 37 °C for 24 h [26]. Subsequently, the cells were treated with rice extracts (0–200 µg/mL) for an additional 24 h.

#### 2.9.2. MTT-Based Cell Viability Test

Treated cells were incubated with 0.5 mg/mL MTT solution (100 μL each well) for 4 h at 37 °C to allow for the reductase-catalyzed formation of purple formazan crystals. The supernatant was then carefully removed, and 200 µL of DMSO was added to dissolve the formazan crystals. The OD was measured at 570 nm with a reference wavelength of 630 nm using a 96-well microplate reader [15]. Cytotoxicity was presented in terms of the percentage of viable cells relative to the control without treatment, which was calculated using Equation (3):

(3) Cell viability (%) = 100 × (OD_570–630 sample_/OD_570–630 control_)

### 2.10. Statistical Analysis

All quantitative experiments were performed in triplicate (*n* = 3) unless otherwise specified. Data were analyzed using the Statistical Package for the Social Sciences (SPSS) Statistics for Windows version 22 Program IBM Corporation, Armonk, NY, USA and presented as mean ± standard deviation values (SD). For comparisons involving more than two groups, significance was determined using the one-way analysis of variance (ANOVA) test, followed by Tukey’s HSD post hoc test to correct for multiple comparisons. For pairwise comparisons, a two-tailed Student’s *t*-test was used. Where multiple hypotheses were tested simultaneously, false discovery rate correction was applied to minimize type I errors. A threshold of *p* < 0.05 was considered statistically significant. When the data did not meet the assumption of normal distribution, nonparametric tests were applied to determine significance.

## 3. Results

### 3.1. Black Rice Preparations and Extracts

**Figure 1:**
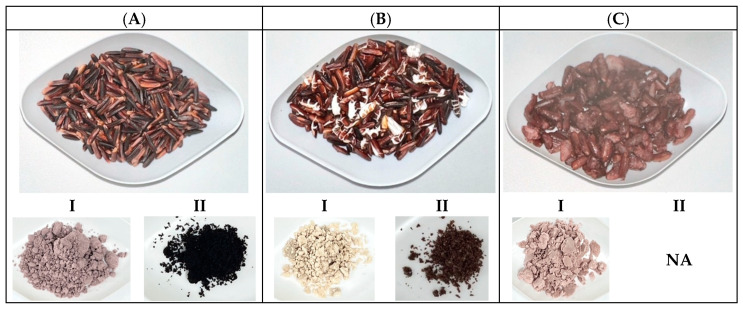
Representative photographs of unpolished Sukhothai fragrant black rice subjected to different processing methods: unprocessed or raw (**A**), popped/puffed (**B**), and streamed cooked (**C**). All rice was then extracted using either hot water (I) or 70% (*v*/*v*) ethanol (II). Each processed rice sample was subsequently extracted using either hot water (I) or 70% (*v*/*v*) ethanol (II), yielding six extracts: URWE, PRWE, CRWE, UREE, PREE, and CREE. Visual inspection indicated that water extracts were generally darker in color than ethanolic extracts across all processing conditions. Extract color intensity decreased progressively from unprocessed to cooked samples. The extraction yields per gram of rice differed among treatments. Water extraction resulted in higher yields than ethanolic extraction for all processing methods. The yields were 64.33 mg/g for URWE, 32.05 mg/g for PRWE, and 13.01 mg/g for CRWE, whereas ethanolic extracts yielded 9.98 mg/g for UREE, 7.60 mg/g for PREE, and 2.54 mg/g for CREE.

Representative photographs of Sukhothai fragrant black rice subjected to three processing methods including unprocessed or raw (A), popped/puffed (B) and steam-cooked (C) are shown in Figure 1. Unprocessed grains retained a deep purple-black coloration, while popped/puffed rice exhibited expanded and porous kernels with a lighter appearance. Steam-cooked rice appeared swollen and glossy.

### 3.2. Effects of Cooking and Processing on Total Phenolic and Total Flavonoid Contents

**Figure 2:**
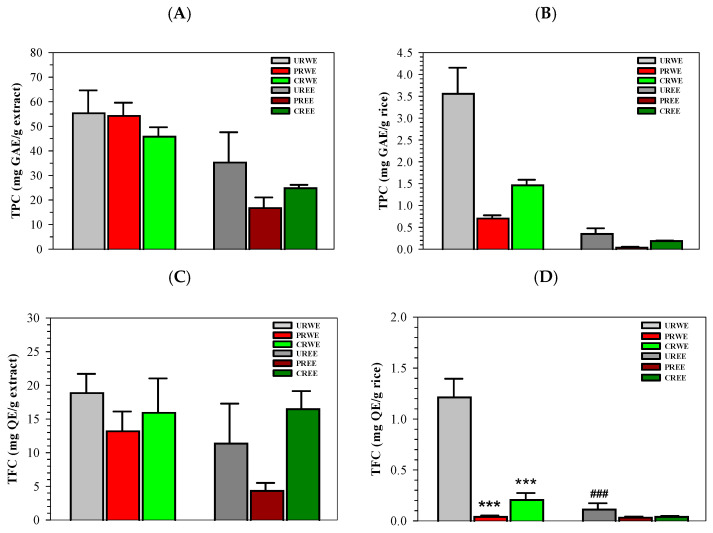
Effects of processing and extraction solvent on TPC and TFC of Sukhothai fragrant black rice extracts. (**A**) TPC expressed as mg GAE/g extract, (**B**) TPC expressed as mg GAE/g rice, (**C**) TFC expressed as mg QE/g extract, and (**D**) TFC expressed as mg QE/g rice. Data represent mean ± SD values obtained from three independent experiments. Accordingly, *** *p* < 0.001 when compared with uncooked rice, ### *p* < 0.001 when compared between the two extractions.

Total phenolic content (TPC) values of the rice extracts are presented in Figure 2A,B. Across all processing conditions, water extracts exhibited higher TPC values than ethanolic extracts. Among water extracts, URWE showed the highest mean TPC value (55.37 ± 13.08 mg GAE/g extract), followed by PRWE (54.32 ± 7.51 mg GAE/g extract) and CRWE (45.84 ± 5.34 mg GAE/g extract). Ethanolic extracts exhibited lower TPC values, with UREE and CREE showing mean values of 35.33 ± 17.36 and 24.89 ± 1.79 mg GAE/g extract, respectively. Statistical analysis using one-way ANOVA indicated no significant differences in TPC among treatments (*p* = 0.234).

Total flavonoid content (TFC) values are shown in Figure 2C,D. Water extracts contained substantially higher flavonoid levels than ethanolic extracts across all processing conditions. URWE exhibited the highest TFC (3.56 ± 0.84 mg QE/g extract), followed by PRWE (1.74 ± 0.24 mg QE/g extract) and CRWE (0.60 ± 0.07 mg QE/g extract). In contrast, flavonoid contents in ethanolic extracts were low, with UREE and CREE exhibiting values of 0.35 ± 0.17 and 0.06 ± 0.00 mg QE/g extract, respectively. Statistical analysis revealed significant effects of processing and extraction solvent on TFC (*p* < 0.001). Thermal treatment and solvent polarity significantly affected flavonoid extraction.

### 3.3. Total Trans-Ferulic Acid Content

**Figure 3:**
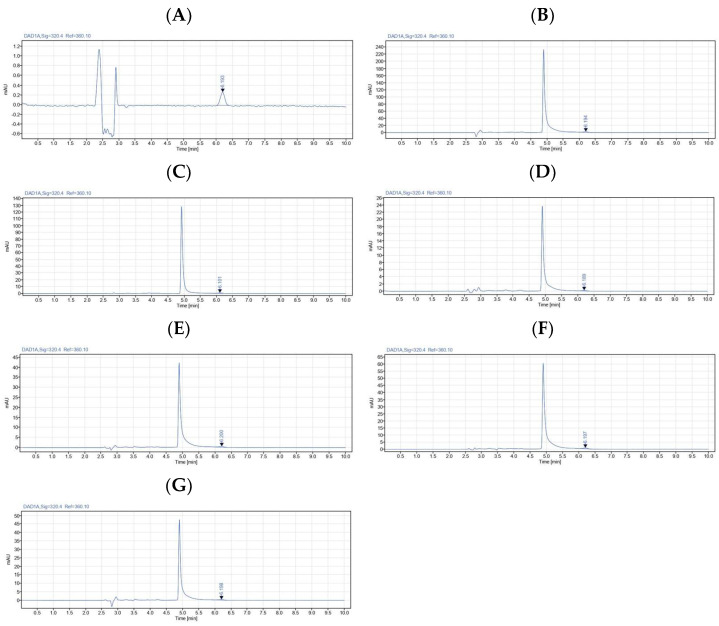
HPLC-DAD profiles of trans-ferulic acid presented in Sukhothai fragrant black rice extracts. (**A**) Authentic trans-ferulic acid standard, (**B**) URWE, (**C**) PRWE, (**D**) CRWE, (**E**) UREE, (**F**) PREE, and (**G**) CREE. Detection was performed at 320 nm.

Representative HPLC-DAD chromatograms of trans-ferulic acid detected in the authentic standard and rice extracts are shown in Figure 3. Distinct peaks corresponding to trans-ferulic acid were detected in all extracts.

Quantitative values of trans-ferulic acid are summarized in Table 1. Among all samples, URWE exhibited the highest trans-ferulic acid content, with a concentration of 174.64 µg/g extract and a total recovery of 11.23 µg/g rice. PRWE and CRWE showed lower concentrations of trans-ferulic acid, with values of 69.98 and 19.68 µg/g extract, respectively. Ethanolic extracts exhibited lower total recoveries per gram of rice but higher concentrations per gram of extract relative to their water-extracted counterparts. Among ethanolic extracts, PREE exhibited the highest trans-ferulic acid concentration (63.86 µg/g extract), followed by UREE (46.72 µg/g extract) and CREE (37.53 µg/g extract).

Taken together, thermal processing (puffing and cooking) diminishes ferulic acid retention. Alternatively, water extraction recovers more overall phenolics due to higher solubility and yield, while ethanol extracts are more concentrated but less abundant. Indeed, unprocessed rice provides the best balance of both extraction yield and ferulic acid content, suggesting that minimal heat treatment preserves antioxidant compounds.

### 3.4. Total γ-Oryzanol Content

**Figure 4:**
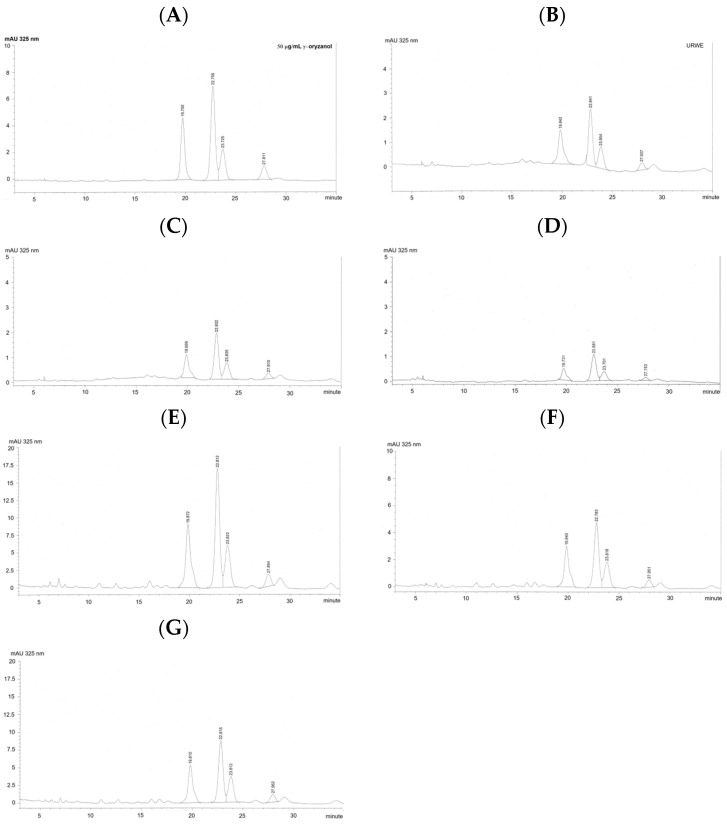
HPLC-DAD chromatographic analysis of γ-oryzanol in Sukhothai fragrant black rice extracts. (**A**) Authentic γ-oryzanol standard, (**B**) URWE, (**C**) PRWE, (**D**) CRWE, (**E**) UREE, (**F**) PREE, and (**G**) CREE. Detection was carried out at 325 nm.

Representative HPLC-DAD chromatograms of γ-oryzanol obtained from the authentic standard and different rice extracts are shown in Figure 4. The γ-oryzanol peaks in all rice extracts exhibited retention times consistent with those of the authentic standard, confirming their identity.

Quantitative analysis revealed variations in total γ-oryzanol content among the different extracts (Table 2). Among the water extracts, PRWE exhibited the highest γ-oryzanol content (1.16 mg/g extract), followed by URWE and CRWE. In contrast, ethanol extracts showed substantially higher γ-oryzanol levels, with CREE exhibiting the highest content (8.85 mg/g extract), followed by PREE and UREE. The observed differences among samples reflect analytical variations under the same experimental conditions rather than biological variability.

### 3.5. Total Anthocyanin Content

**Figure 5:**
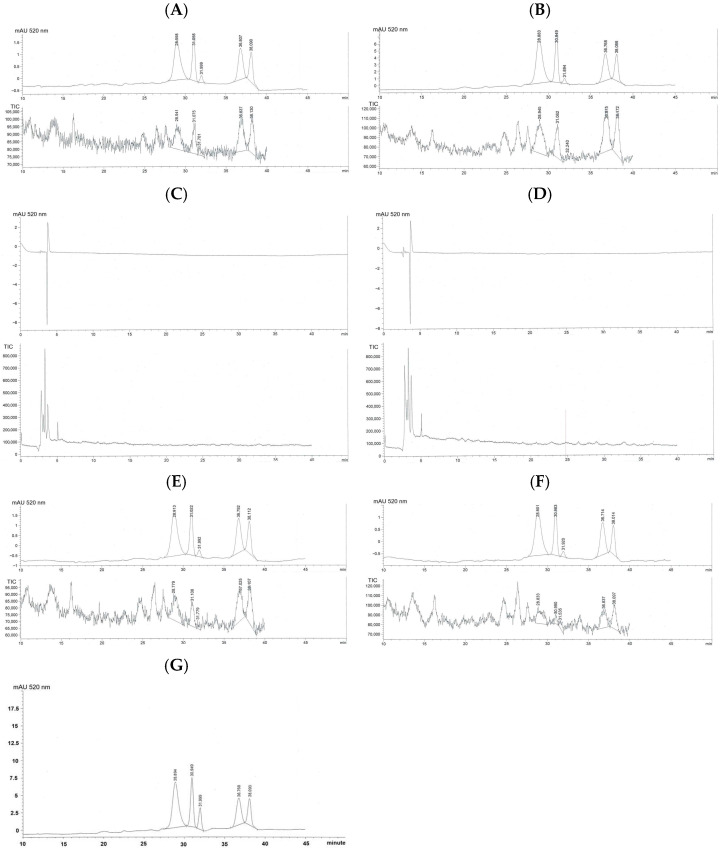
HPLC-ESI-MS analysis of anthocyanins in the authentic standards (**A**), URWE (**B**), PRWE (**C**), CRWE (**D**), UREE (**E**), PREE (**F**), and CREE (**G**).

Figure 5 presents the HPLC-ESI-MS chromatographic profiles of authentic anthocyanin standards and various rice extracts. The authentic standards (Figure 5A) show well-resolved peaks at characteristic retention times, confirming the selectivity and accuracy of the analytical method. Comparable retention times observed in URWE and PRWE chromatograms (Figure 5B,C) indicate the presence of identical anthocyanin compounds in these samples. In contrast, CRWE, UREE, and PREE chromatograms (Figure 5D–F) exhibit either very low-intensity peaks or non-detectable signals, suggesting anthocyanin concentrations below the quantification limit. The reduced peak intensity and peak number in ethanol extracts compared to water extracts indicate a statistically meaningful reduction in anthocyanin extraction efficiency when ethanol is used as the solvent. Overall, the results demonstrate a clear qualitative difference in anthocyanin profiles among rice varieties and extraction methods.

Also reported in Table 3, PRWE exhibited the highest mean concentrations of all quantified anthocyanins, with total anthocyanin glucosides reaching 3377.49 µg/g extract, which is approximately five-fold higher than URWE (672.22 µg/g extract). This large magnitude difference suggests a strong effect size attributable to rice variety and extraction conditions. Among individual compounds, cyanidin-3-glucoside consistently represents the dominant anthocyanin, accounting for the largest proportion of total anthocyanins across detectable samples. This indicates a skewed distribution of anthocyanin composition, where one compound contributes disproportionately to total pigment content. Several samples (CRWE, UREE, and PREE) report values below the limit of quantification (<0.20 µg/g extract) for most anthocyanins. From a statistical perspective, these values indicate non-significant concentrations, reinforcing the conclusion that these extracts contain negligible anthocyanin levels relative to PRWE and URWE. When normalized to rice weight (µg/g rice), the same trend is preserved, confirming that differences are not solely due to extraction yield, but reflect true biological variation in anthocyanin content among rice samples.

### 3.6. Antioxidant Activity

**Figure 6:**
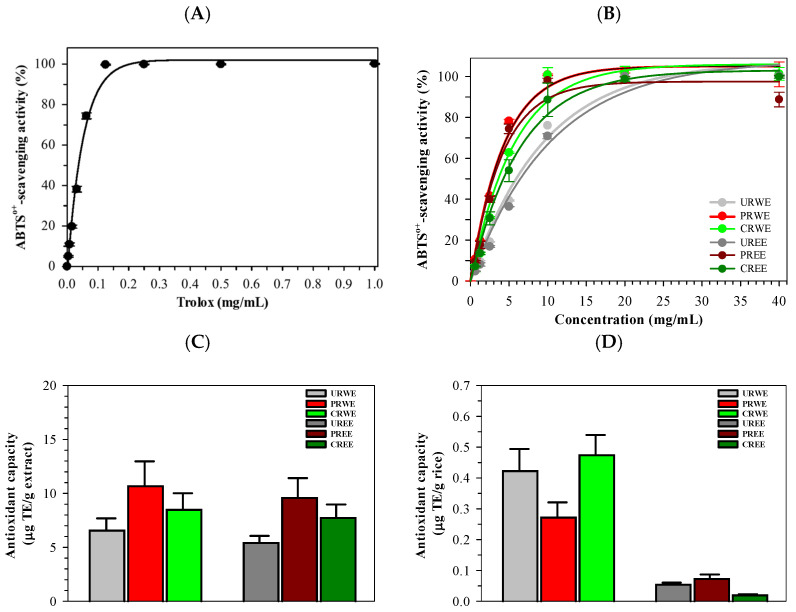
Inhibitory effect of ABTS^•+^ generation by Trolox (**A**) and rice extracts (**B**), and antioxidant activities expressed per g (**C**) and per g of rice (**D**). Data represent mean ± SEM values of three independent experiments.

The antioxidant activities of rice extracts were evaluated using ABTS and DPPH radical scavenging assays. Trolox as well as all rice extracts exhibited concentration-dependent radical scavenging activity (Figure 6A,B). For the ABTS assay, Trolox showed the strongest scavenging activity, with an IC_50_ value of 0.06 ± 0.00 mg/mL (Table 4). Among rice extracts, water extracts exhibited lower IC_50_ values than ethanolic extracts. URWE, PRWE, and CRWE showed IC_50_ values of 2.81 ± 0.15, 3.81 ± 0.18, and 3.66 ± 0.20 mg/mL, respectively. Ethanolic extracts exhibited higher IC_50_ values, indicating lower ABTS scavenging activity. Antioxidant capacity expressed as Trolox equivalents (TE)/g of extract is shown in Figure 6C. Among water extracts, PRWE and URWE exhibited higher antioxidant capacity than CRWE. All ethanolic extracts exhibited lower antioxidant capacity than water extracts. When antioxidant activity/g of rice was expressed (Figure 6D), CRWE exhibited higher values than URWE and PRWE, while ethanolic extracts exhibited minimal activity.

**Figure 7:**
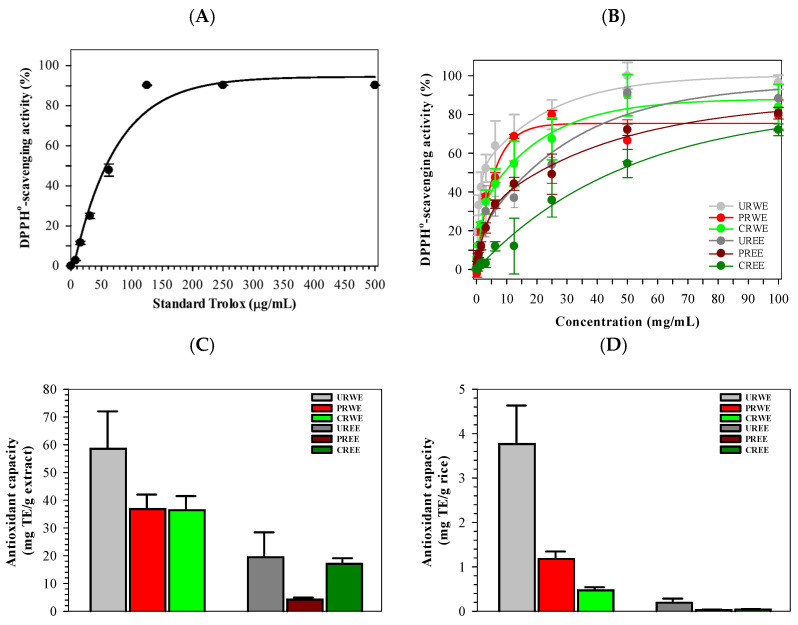
Inhibitory effect of DPPH^•^ generation by Trolox (**A**) and rice extracts (**B**), and antioxidant activities per g (**C**) and g of rice (**D**). Data represent mean ± SEM values of three independent experiments.

Similar trends were observed in the DPPH assay. Trolox showed IC_50_ value of 67.77 μg/mL (Figure 7A), URWE exhibited the lowest IC_50_ value among rice extracts (2.53 ± 0.13 mg/mL), followed by PRWE (6.04 ± 0.25 mg/mL) and CRWE (9.16 ± 0.32 mg/mL). Ethanolic extracts showed higher IC_50_ values, with CREE exhibiting the weakest DPPH scavenging activity (45.42 ± 1.21 mg/mL) (Figure 7B). Antioxidant capacity expressed per gram of extract and per gram of rice is shown in Figure 7C,D and Table 4.

### 3.7. Huh7 Cell Viability

**Figure 8:**
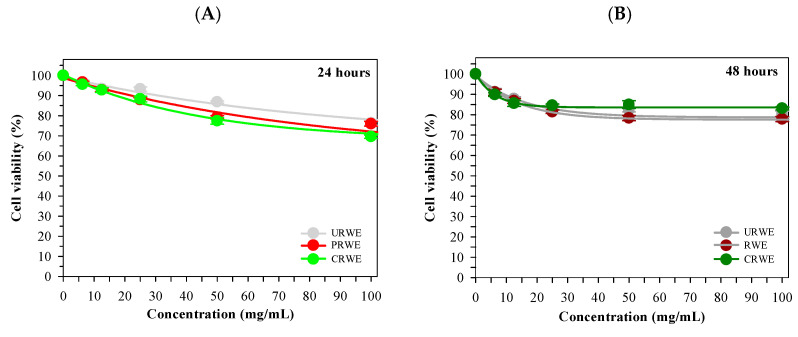
Viability of Huh7 cells treated with rice extracts for 24 h (**A**) and 48 h (**B**). Data represent mean ± SD values obtained from three independent experiments.

The effects of rice extracts on Huh7 cell viability are shown in Figure 8. After 24 h of treatment, cell viability remained relatively high for water extracts (URWE, PRWE, and CRWE) across all tested concentrations. In contrast, ethanolic extracts (UREE, PREE, and CREE) resulted in greater reductions in cell viability. After 48 h of treatment, decreases in cell viability were more pronounced for ethanolic extracts, particularly UREE and PREE, compared with water extracts. Water extracts exhibited comparatively smaller reductions in viability at the same concentrations and time points.

## 4. Discussion

This study systematically evaluated the effects of thermal processing and extraction solvent on the phytochemical composition, antioxidant capacity, and cytotoxic activity of Sukhothai fragrant black rice. By comparing unprocessed, popped/puffed, and steam-cooked rice extracted with hot water or 70% ethanol, the results provide clear evidence that both processing intensity and solvent polarity play decisive roles in determining extract yield, phenolic composition, and biological activity.

Water extraction consistently produced higher extract yields than ethanolic extraction, indicating that the majority of extractable constituents in black rice are polar compounds. These findings agree with previous reports demonstrating that anthocyanins and phenolic acids—major contributors to the antioxidant capacity of pigmented rice—exhibit greater solubility in aqueous media than in ethanol. In contrast, ethanolic extraction yielded smaller quantities of extract but with higher concentrations of less-polar phenolic compounds, consistent with solvent polarity–dependent selectivity [27,28]. Thermal processing significantly influenced extract yield and composition. Steam cooking resulted in the lowest extraction yields, whereas popping/puffing produced intermediate yields between unprocessed and cooked rice. These changes likely reflect alterations in the rice matrix during heating. High-moisture heat associated with cooking promotes starch gelatinization and protein denaturation, which can entrap phenolic compounds and reduce their extractability. In contrast, the rapid dry-heat expansion during puffing disrupts cellular structures and partially exposes bound phenolics, although exposure to high temperature may simultaneously degrade heat-labile compounds [29,30]. These structural and compositional changes were reflected in both extract yields and color intensity. Total phenolic and flavonoid contents were strongly influenced by extraction solvent and processing. Water extracts consistently exhibited higher TPC and TFC values than ethanolic extracts, confirming that polar phenolics dominate the antioxidant profile of black rice [28,31]. Among processing treatments, unprocessed rice retained the highest phenolic and flavonoid contents, while steam cooking resulted in notable reductions. These observations are consistent with previous studies reporting thermal degradation, oxidation, and leaching of phenolics during cooking [32,33,34]. Popping/puffing preserved intermediate phenolic levels, supporting the concept that dry-heat expansion can enhance extractability of some bound phenolics while still causing partial degradation of thermolabile compounds [29,30].

Quantitation of trans-ferulic acid demonstrated the effects of processing and solvent polarity. Unprocessed rice water extract exhibited the highest total recovery of trans-ferulic acid, indicating that minimal thermal treatment best preserves this compound. Puffing and steam cooking markedly reduced ferulic acid recovery, consistent with previous reports showing that hydroxycinnamic acids undergo thermal degradation, oxidation, or matrix entrapment during heating [10,35]. Ethanolic extracts contained higher concentrations of ferulic acid per gram of extract but lower overall recovery per gram of rice, reflecting selective solubility rather than improved preservation. These findings highlight the importance of distinguishing between compound concentration and total recovery when evaluating extraction efficiency. γ-Oryzanol, a mixture of ferulic acid esters of phytosterols and triterpene alcohols, is a major bioactive compound in rice and rice-derived products and is widely recognized for its antioxidant and health-promoting properties. In this study, total γ-oryzanol in different rice extracts was successfully quantified using an HPLC-DAD method, with chromatographic profiles and retention times consistent with those of the authentic standard. Similar analytical performance has been reported in previous studies employing reversed-phase HPLC with detection at approximately 325 nm, corresponding to the absorbance maximum of ferulate moieties [20,36]. Substantial variation in γ-oryzanol content was observed among the extracts, with ethanol extracts exhibiting markedly higher levels than water extracts. This finding is consistent with the hydrophobic nature of γ-oryzanol, which limits its solubility in aqueous media. Organic solvents such as ethanol have been shown to enhance the extraction efficiency of γ-oryzanol from rice matrices and rice bran oil, supporting the extraction trends observed in this study [37,38]. From a mechanistic perspective, the antioxidant activity of γ-oryzanol is primarily attributed to the ferulic acid moiety, which can donate hydrogen atoms or electrons to neutralize free radicals and inhibit lipid peroxidation. In addition, the steryl and triterpenyl components of γ-oryzanol contribute to membrane stabilization and protection against oxidative damage. These combined mechanisms underpin the reported antioxidant, anti-inflammatory, and lipid-lowering effects of γ-oryzanol in food and biological systems [39,40]. Although the present study was conducted as a single analytical experiment and the data are reported descriptively, the observed trends align well with previously published literature. The results highlight the importance of solvent selection for maximizing γ-oryzanol recovery and confirm the suitability of ethanol-based extraction coupled with HPLC-DAD analysis for comparative profiling of γ-oryzanol in rice extracts. Future studies incorporating replicate analyses and functional assays would further strengthen the linkage between γ-oryzanol content and antioxidant activity.

HPLC-ESI-MS analysis of anthocyanin in rice extracts demonstrates clear differences driven by rice variety and extraction conditions. Such variation is consistent with extensive prior work showing that pigmented rice (black/purple) accumulates substantially higher anthocyanins than non-pigmented rice, largely in the bran/pericarp layer [35,41,42]. In the current dataset, PRWE exhibited markedly higher total anthocyanin glucosides than other extracts, indicating a strong varietal effect. Similar cultivar-dependent variation has been widely reported and is typically attributed to genetic regulation of anthocyanin biosynthesis and pigment deposition in the outer grain layers [42,43]. Herein, the qualitative chromatographic evidence supports this conclusion, as pigmented rice water extracts show clear peaks corresponding to anthocyanins, while several other extracts display minimal or non-quantifiable signals. Across detectable samples, cyanidin-3-glucoside was the dominant compound, with other anthocyanins present at lower levels (e.g., peonidin derivatives and minor glycosides). This pattern agrees strongly with the literature: multiple studies using HPLC-DAD and HPLC-MS methods identify cyanidin-3-glucoside as the major anthocyanin in black/pigmented rice, often comprising the majority fraction, with peonidin-3-glucoside as the next most abundant component [6,44,45]. Likewise, pigmented rice describes cyanidin-3-glucoside and peonidin-3-glucoside as principal anthocyanins associated with both color and bioactivity [35]. Extraction solvent effects in the present results are also consistent with published evidence. Water-based (often acidified) systems frequently enhance recovery of polar anthocyanin glycosides, whereas ethanol-based extraction outcomes can vary depending on ethanol concentration, acidity, and processing conditions, influencing solubility and stability [43,46]. The frequent <LOQ values in several ethanol extracts may therefore reflect reduced extraction efficiency and/or stability differences under the tested conditions. The magnitude and consistency of differences; especially the substantially higher totals in PRWE, support biologically meaningful variation consistent with prior rice anthocyanin profiling studies [43,44,45].

The antioxidant activities measured by ABTS and DPPH assays closely paralleled trends in phenolic and flavonoid contents. Water extracts, particularly those from unprocessed and popped rice, exhibited stronger radical-scavenging activity than ethanolic extracts. This strong correspondence supports previous findings that phenolic acids and flavonoids are primary contributors to antioxidant activity in rice [3,10,47]. The reduced antioxidant activity observed in cooked samples reflects the sensitivity of these compounds to prolonged heat exposure [10,35]. Differences between ABTS and DPPH results likely reflect their distinct reaction mechanisms and sensitivities toward hydrophilic versus lipophilic antioxidants [48].

The cytotoxicity results demonstrated clear solvent- and time-dependent effects on Huh7 hepatocellular carcinoma cells. Ethanolic extracts, particularly those derived from unprocessed and popped rice, exhibited greater reductions in cell viability than water extracts, especially after prolonged exposure. This enhanced cytotoxicity may be attributed to the enrichment of less-polar bioactive compounds in ethanolic extracts, such as flavonoid aglycones or sterol-related constituents, that may exhibit higher cellular permeability and intracellular activity [49,50]. In contrast, water extracts rich in polar phenolics and anthocyanin glycosides exhibited milder effects, consistent with their lower membrane permeability. Thermal processing further influenced cytotoxic potential, with extracts from unprocessed and popped rice demonstrating stronger activity than those from cooked rice, suggesting degradation or loss of cytotoxic constituents during steam cooking [10,35].

Overall, this study highlights that extraction solvent and processing method must be carefully selected depending on the intended application of black rice extracts. Aqueous extraction of minimally processed rice is optimal for maximizing phenolic recovery and antioxidant capacity, while ethanolic extraction enhances biological potency despite lower yields. These findings are particularly relevant for the development of functional foods and nutraceutical products derived from pigmented rice.

Several limitations should be acknowledged. This study focused primarily on trans-ferulic acid as a representative phenolic compound, while other important bioactives such as anthocyanins, protocatechuic acid, and γ-oryzanol were not quantitatively assessed. Only a single set of popping and cooking conditions was examined, and variations in temperature, pressure, or duration may yield different outcomes. In addition, antioxidant and cytotoxic assays provide valuable mechanistic insights but do not fully represent in vivo bioavailability or metabolism. Future studies incorporating advanced metabolomic profiling, kinetic degradation analyses, and in vivo validation would further strengthen understanding of how processing influences the health-promoting potential of black rice.

Combining sensory evaluation and product formulation could also support the development of functional black rice products that balance nutritional and consumer acceptability. Finally, subsequent research studies should explore encapsulation or fermentation technologies to enhance the bioavailability and stability of rice-derived phenolic compounds during processing and storage.

## 5. Conclusions

This study has revealed that both thermal processing and solvent extraction significantly influences the phytochemical composition, antioxidant capacity and cytotoxicity of Sukhothai fragrant black rice. Unprocessed rice retained the highest levels of trans-ferulic acid, total phenolics, flavonoids and anthocyanins, resulting in superior antioxidant activity, whereas popping and steaming led to notable reductions due to heat-induced degradation. Hot-water extraction yielded higher extract recoveries and more effectively preserved polar antioxidant compounds, while 70% ethanolic extraction produced lower yields but enriched less-polar bioactive constituents associated with enhanced cytotoxic effects against Huh7 cells. Overall, minimal thermal processing combined with aqueous extraction is optimal for maximizing antioxidant potential, whereas ethanolic extraction may be suitable for applications targeting biological activities. These findings provide practical guidance for selecting processing and extraction strategies to enhance the nutritional and functional value of black rice for food and nutraceutical applications.

## Figures and Tables

**Table 1:** Absolute values of total trans-ferulic acid content in URWE, PRWE, CRWE, UREE, PREE, and CREE.

Sample	Trans-Ferulic Acid		
	PA (320 nm)	(μg/g)	(μg/g of Rice)
Trans-ferulic acid (0.1 μg/mL)	2.69	-	-
URWE	1.56	174.64	11.23
PRWE	6.45	69.98	2.24
CRWE	17.02	19.68	0.26
UREE	2.83	46.72	0.47
PREE	5.25	63.86	0.49
CREE	3.68	37.53	0.10

**Table 2:** Absolute values of total γ-oryzanol content in rice extracts.

Sample	Total γ-Oryzanol	
	(mg/g Extract)	(μg/g Rice)
Total γ-oryzanol (50 μg/mL)	-	-
URWE	0.86	55.32
PRWE	1.16	37.18
CRWE	0.39	5.07
UREE	0.91	9.08
PREE	3.63	27.59
CREE	8.85	22.48

**Table 3:** Absolute values of different anthocyanin compounds in rice extracts.

Time	Anthocyanins	Sample					
(min)		URWE	PRWE	CRWE	UREE	PREE	CREE
28.843	Cyanindin-3-glucoside						
	(μg/g extract)	248.00	1629.05	<0.20	<0.20	226.52	389.31
	(μg/g rice)	15.87	52.13	NA	NA	1.72	0.99
30.949	Keracyanin-3-rutinoside						
	(μg/g extract)	90.01	584.37	<0.20	<0.20	78.97	133.23
	(μg/g rice)	5.76	187.00	NA	NA	0.60	0.34
31.999	Callistephin glucoside						
	(μg/g extract)	15.58	62.98	<0.20	<0.20	12.83	16.18
	(μg/g rice)	1.00	2.02	NA	NA	0.10	0.04
36.768	Peonidin glucoside						
	(μg/g extract)	98.76	508.22	<0.20	<0.20	89.13	138.30
	(μg/g rice)	6.32	16.26	NA	NA	0.68	0.35
38.090	Malvidine-3-galactoside						
	(μg/g extract)	87.40	443.09	<0.20	<0.20	74.12	100.98
	(μg/g rice)	5.59	14.18	NA	NA	0.56	0.26
	Total anthocyanin glucoside						
	(μg/g extract)	672.22	3377.49	2.59	2.59	576.85	758.12
	(μg/g rice)	43.22	108.08	0.03	0.03	4.38	1.92

Abbreviation: NA = not available.

**Table 4:** IC_50_ Values (mean ± SD) of Trolox, rice water extracts, and ethanolic extracts determined by the ABTS and DPPH assays.

Sample	IC_50_ Values (mg/mL)	
	ABTS Method	DPPH Method
Trolox	0.06 ± 0.00 ^a^	0.07 ± 0.00 ^a^
URWE	2.81 ± 0.15 ^b^	2.53 ± 0.13 ^b^
PRWE	3.81 ± 0.18 ^b,c^	6.04 ± 0.25 ^c^
CRWE	3.66 ± 0.20 ^b,c^	9.16 ± 0.32 ^d^
UREE	6.56 ± 0.29 ^c,d^	17.35 ± 0.71 ^e^
PREE	4.22 ± 0.24 ^c,d^	20.47 ± 0.80 ^e^
CREE	2.97 ± 0.12 ^b^	45.42 ± 1.21 ^f^

^a–f^ Denotes significant differences at *p* < 0.05 (Tukey’s HSD) in which ^a^ represents the highest activity and ^f^ represents the lowest activity. Lower IC_50_ values indicate stronger antioxidant activity.

## Data Availability

The original contributions presented in this study are included in the article. Further inquiries can be directed to the corresponding author.

## Abbreviations

The following abbreviations are used in this manuscript:

ABTS	2,2′-Azino-bis(3-ethylbenzothiazoline-6-sulfonic acid)
CREE	Cooked Rice Ethanolic Extract
CRWE	Cooked Rice Water Extract
DI	Deionized Water
DMEM	Dulbecco’s Modified Eagle Medium
DMSO	Dimethyl Sulfoxide
DPPH	2,2-Diphenyl-1-picrylhydrazyl
FA	Ferulic Acid
GA	Gallic Acid
GAE	Gallic Acid Equivalent
HPLC	High-Performance Liquid Chromatography
HPLC-DAD	High-Performance Liquid Chromatography with Diode Array Detector
IC_50_	Half-Maximal Inhibitory Concentration
MTT	3-(4,5-Dimethylthiazol-2-yl)-2,5-diphenyltetrazolium Bromide
OD	Optical Density
PRWE	Popped Rice Water Extract
PREE	Popped Rice Ethanolic Extract
PTFE	Polytetrafluoroethylene
Q	Quercetin
QE	Quercetin Equivalent
TE	Trolox equivalent
TFC	Total Flavonoid Content
TPC	Total Phenolic Content
URWE	Unprocessed Rice Water Extract
UREE	Unprocessed Rice Ethanolic Extract
UV–Vis	Ultraviolet–Visible Spectrophotometer
